# The Nurse-Apprentice

**Published:** 1920-08-21

**Authors:** 


### THE MATRONS' AND SISTERS' DEPARTMENT.

### THE NURSE-APPRENTICE.

Thousands of potential good nurses are lost to the profession between the ages of eighteen and twenty. The crying need in the nursing world is to bridge over these years and set nursing before girls leaving school as a practicable proposition, instead of as a far-off dream.

The first step towards this desirable consummation has been attained in the shortening of the age at which probationers are received. The hard-and-fast rule excluding everyone below twenty-three has long passed into oblivion; it is as extinct as the practice of the well-known matron in days gone by who resorted in despair to the method of measuring applicants and rejecting off-hand all above or below her too literal standard. Experiences in war hospitals have showed once and for all the fitness of girls under twenty to do good service with the sick, and there are few matrons at the present time who would look askance at candidates mentally and physically well balanced solely because of their youth.

The new notion of the nurse-apprentice, as explained in the pages of the *College Bulletin* for July, rests on the assumption that the actual nursing career may commence before the age of twenty. "The nurse-apprentice," we learn, "is to be taught on modernised lines. Straight from school at eighteen, she is to be passed through a preparatory course lasting one year, which will embrace the theoretical knowledge with which the efficient future combatant of illness and disease must be equipped, whether eventually she branches out into specialised sick nursing in an institution, into general sick nursing in people's homes, into maternity care or child welfare, or into what is destined to be the most important of all—the preventive work of a health official. Then she is to do three years' practical work in a hospital." We further learn that the King's College for Women has formulated a scheme which may bring the year's work prior to hospital training in touch with the necessary preparation for the work of the health visitor.

The whole conception of the nurse's career is considered for the first time in relation to public and family requirements. The fatal error of allowing the potential nurse on leaving school to drift into some other career which will probably absorb her altogether is avoided. But there is still much to be done before any considerable number of young women can take advantage of the scheme. It is thought probable that many girls will require grants to maintain them while in training. But the practice of grants during training, though found absolutely necessary in the case of elementary teachers, may easily prove a stumbling-block. It is doubtful how far the Ministries of Health and Education might be able to develop nurse-training on these lines. Taking into consideration the large number of probationers needed in the hospitals and infirmaries, it must appear that only the smallest percentage of their number could be affected by a system of grants in aid, at any rate in the present state of public finances. From financial reasons alone, we deem it essential that provision should be made for providing the course of study preliminary to hospital-training in centres where they would be accessible to young women living at home.

No one doubts the advantage of collegiate residence. But the training-schools, in which the nurse-apprentice will pass three years, should afford this element in the nurse's course. For the year following school external teaching should suffice, and could quite easily be placed within the reach of very large numbers of pupils. The cost of maintaining scholars at the King's College for Women is estimated at £150 a year. The fees for attendance at lectures, classes, and demonstrations would not exceed from £30 to £45. The cost of equipping collegiate establishments to meet a great rush of nursing apprentices in London and the provinces would be incalculable, and must certainly delay the wished-for reform indefinitely. The cost of running a course for extern nurse-apprentices in organised University centres in various parts of the country would be negligible, for the lecturers' fees, etc., would be met out of the students' payments.

We look to the College for a clear pronouncement on the vexed question of a curriculum for such a year's course as has been indicated. The writer in the *Bulletin* is guarded on this subject. She intimates that the apprentice would learn "what normal conditions should be in a healthy community, what the standard normal structure and functions of a human being are in health." If these lines are followed, it may be safely prophesied that not only women who desired to become nurses, but many who merely desired to equip themselves for the battle of life would take advantage of the course.

